# Colorectal Cancer—The “Parent” of Low Bowel Obstruction

**DOI:** 10.3390/medicina59050875

**Published:** 2023-05-02

**Authors:** Valentin Titus Grigorean, Anwar Erchid, Ionuţ Simion Coman, Mircea Liţescu

**Affiliations:** 1 General Surgery Department, “Carol Davila” University of Medicine and Pharmacy, 37 Dionisie Lupu Street, 020021 Bucharest, Romania; grigorean.valentin@yahoo.com (V.T.G.); ionut.coman@umfcd.ro (I.S.C.); mircealitescu@gmail.com (M.L.); 2 General Surgery Department, “Bagdasar-Arseni” Clinical Emergency Hospital, 12 Berceni Road, 041915 Bucharest, Romania; 3 General Surgery Department, “Sf. Ioan” Clinical Emergency Hospital, 13 Vitan-Bârzeşti Road, 042122 Bucharest, Romania

**Keywords:** cancer, colorectal, obstruction

## Abstract

*Introduction*: Despite the improvement of early diagnosis methods for multiple pathological entities belonging to the digestive tract, bowel obstruction determined by multiple etiologies represents an important percentage of surgical emergencies. *General data*: Although sometimes obstructive episodes are possible in the early stages of colorectal cancer, the most commonly installed intestinal obstruction has the significance of an advanced evolutionary stage of neoplastic disease. *Development of Obstructive Mechanism*: The spontaneous evolution of colorectal cancer is always burdened by complications. The most common complication is low bowel obstruction, found in approximately 20% of the cases of colorectal cancer, and it can occur either relatively abruptly, or is preceded by initially discrete premonitory symptoms, non-specific (until advanced evolutionary stages) and generally neglected or incorrectly interpreted. Success in the complex treatment of a low neoplastic obstruction is conditioned by a complete diagnosis, adequate pre-operative preparation, a surgical act adapted to the case (in one, two or three successive stages), and dynamic postoperative care. The moment of surgery should be chosen with great care and is the result of the experience of the anesthetic-surgical team. The operative act must be adapted to the case and has as its main objective the resolution of intestinal obstruction and only in a secondary way the resolution of the generating disease. *Conclusions*: The therapeutic measures adopted (medical-surgical) must have a dynamic character in accordance with the particular situation of the patient. Except for certain or probably benign etiologies, the possibility of colorectal neoplasia should always be considered, in low obstructions, regardless of the patient’s age.

## 1. Introduction

Despite the improvement of early diagnosis methods for multiple pathological entities belonging to the digestive tract, bowel obstruction determined by multiple etiologies represents an important percentage of surgical emergencies, accounting for approximately 20–30% of the cases diagnosed with acute abdomen [1]. Efforts made to identify and treat inflammatory diseases of the small intestine and colon, diagnosis of colic and rectal neoplasms in early stages, or the surgical resolution of parietal defects in uncomplicated stage have brought improvements regarding the precipitation of an obstructive episode of various etiologies [2].

This research focuses on the determinism, the physiological mechanisms, and the treatment particularities of obstructive colorectal cancer, an entity representing 60–80% of low bowel obstructions [3,4]. 

## 2. General Data

Intestinal obstruction is commonly found in surgical services, as a stand-alone entity (with impressive etiological, pathogenic, and topographic diversity), or as an epiphenomenon of other medical or surgical conditions (basal pneumonia, acute appendicitis, acute pancreatitis, etc.). Although the clinic is generally sufficient for positive and topographic diagnosis, the etiological and pathogenic details cannot be clearly outlined, losing their specificity due to an intricate clinic with a spectacular dynamic of the suggestive elements for certain generating causes. With the exception of obstructions with ischemic mechanism from the beginning (complicated parietal defects, intestinal intussusceptions and volvulus, internal hernias, etc.), those of the simple obstructive type present a clinical, dynamic mosaic, which frequently fails the attempts of systematization, creating taxonomic controversies, but justifies hydro-electrolytic, acid-base and nutritional rebalancing measures and finally the surgical procedure [5].

The contribution of paraclinical and laboratory investigations is extremely useful, but even in these conditions a lot of cases remain etiologically obscure [3,6]. Clinical aspects are even more nuanced when the obstructive accident occurs after surgery. The interplay of anatomical and functional causative elements, as well as clinical atypia, explains the diagnostic difficulties and medical-surgical treatment that is difficult to standardize [7].

A serious clinical entity by itself, intestinal obstruction can also be complicated (abscessed tumors, bleeding, diastatic perforations, etc.) which produces an exponential worsening generating mortality rates comparable to severe digestive bleeding, severe pancreatitis, or major sepsis [8].

Distal bowel obstructions (colorectal) have a simple obstruction (except volvulus) as their established mechanism. Symptoms are more indefinite, and the worsening of the general condition occurs more slowly. These so-called “advantages” are nullified by factors, such as age, etiology (often malignant), and multiple complications (most commonly septic). The decompressing factor that the small intestine can have in distal obstructions can be canceled by a pressure-competent ileocecal valve, transforming the colon into a closed, under-pressure loop (double obturated) [9]. Massive and polymorphic bacterial translocation, colic perforations (adjacent to the tumor or diastatic), or diffuse parietocolic necrosis are factors that can lead to rapid, sometimes irreversible worsening. The massive release of endotoxins and digestive enzymes in conditions of compromised mucous-epithelial barrier and microbial populations with exacerbated pathogenicity, explains the initiation of harmful systemic effects, even in the absence of intestinal perforation. The existence and severity of this pathogenic link are confirmed by toxico-septic phenomena being maintained even after the surgical removal of the lesion that generated bowel obstruction [10,11].

Ischemic-type rheologic changes have multiple pathogenies. The cumulative effect of colic parietal vessel elongation (as a result of progressive intestinal distension), the ischemia produced by direct tissue pressure (brides or lateral obstructions), extensive hemorrhagic intraparietal changes (as a result of the rupture of the vessels in the colic wall), or hypovolemic parietal hypoxia contribute to the premature alteration of the mucous-epithelial barrier, and then to colic perforation [3]. 

Massive fluid-electrolyte intersectoral redistributions, with the formation of the IIIrd surgical space, associated with hydro-electrolyte losses through vomiting, contribute to the establishment of the dysvolemic status (up to critical hypovolemia), which in association with the installed toxic-septic status, represent a powerful pathogenic association [12].

The first pathogen that occurs is the impairment of lumen freedom, with upstream storage of gas and stercoral content. Secondarily, enteral motility disorders are installed as a result of cholic distension and episodic appearance of hyperperistaltism for evacuation purposes (“fighting colic’’). The tertiary element that occurs is the modification of intestinal wall viability with the addition of infectious factor (tumor abscess) or juxtatumoral or diastatic colic perforations. Low digestive malignancy, along with progressive lumen obstruction, can precipitate the obstructive episode through other mechanisms: invagination of pediculate tumors, extrinsic parietal invasions, obstructive carcinomatosis, the association of ischemic colic sufferings, etc. From this perspective, mechanical intestinal occlusion presents an initiator of pathogenic mechanisms (the obstacle in the colic lumen) and a systemic resonator (the set of general changes) that worsens itself by dysvolemia and sepsis [13].

Although sometimes obstructive episodes are possible in the early stages of colorectal cancer, the most commonly installed intestinal obstruction has the significance of an advanced evolutionary stage of neoplastic disease. Malignant colorectal obstructions generally evolve with an afebrile state. “Warm” obstructions suggest the appearance of septic, ischemic or co-existence of multiple metastases with hyperpyrexia, accentuated in the context of intersectoral dehydration [14].

A therapeutic attitude in confirmed or intuited non-ischemic cases begins with measures aimed at hydro-electrolyte, acid-base, metabolic rebalancing, and measures to release the intestinal territory proximal to the stenosis through sustained digestive aspiration (less effective in distal obstacles) and retrograde rectocolic lavage. The rhythm and duration of these measures remain an equation with multiple unknowns and traps, but aim at reconfiguring the general state, correcting the installed imbalances (most often partial), impregnating with antibiotic and possibly anticoagulant, cardiac tonic, etc. [13].

Depending on the specific situation, these measures can be adopted regulated or ultra-quickly. In the case of a favorable response, this period can be extended, hoping for complete release, which would allow a safer and more comfortable surgical act, in elective conditions. Failure to respond requires emergency surgery. The surgical attitude cannot be standardized considering the multitude of factors involved, but it ranges from large-scale surgical interventions aimed at solving the generating injury (tumor) as well as the complication (obstruction), to minimal surgical gestures that can contribute to the resolution of the obstruction (cecostomy) [15,16].

Although there is no unity of opinion regarding surgical strategies depending on topography, for the right colon, the right hemicolectomy followed by ileo-transverse anastomosis maintains a leading position, while for tumors located under the splenic flexure of the colon, the interventions in two or three times with different types of stomas upstream are valid, practiced for reasons of safety or necessity.

Quality pre-operative preparation, a well-conducted and adapted surgical act and meticulous postoperative care can bring good results with the resolution of the underlying disease (neoplasm) and its complication (obstruction). However, this morbid association represents a severe pathological circumstance, in front of which optimism must remain moderate and circumspect [17,18].

## 3. Determinism of Colorectal Cancer 

Although there are embryological, anatomical, histological, and functional differences between colic and rectal locations respectively, we note the existence of some common elements between the neoplasms of these segments.

### 3.1. Embryological Factor

The middle portion of the primitive intestine (mesenteron) generates the upper structures of the digestive tract (duodenum, jejunum, ileum), but also cecum, appendix vermiform, ascending colon, and right half of the transverse colon. The metenteron (embryonic posterior intestine) will develop the distal half of the transverse colon, descendant, sigmoid, rectum and upper portion of the anal canal. Studies on the different embryological origins of malignant colorectal segments suggest possible connections between embryology and carcinogenesis, this area of research being of high interest and requiring further studies [19]. 

### 3.2. Genetic Factor

The involvement of genetic factors with autosomal dominant transmission was confirmed with the identification of hereditary nonpolyposis neoplasm (Lynch Syndrome I and II) and adenomatous familial polyposis [20,21].

In addition, it is important to mention the adenoma-carcinoma sequence, which is defined by a set of recurrent driver mutations in a series of genes (KRAS, APC, SMAD4, TP53) that accumulate in the process of adenoma formation and progression to sporadic colorectal cancer [22].

### 3.3. Histological Factor

The entire colon and rectum above the pectinated line is lined with one layered columnar epithelium. Below this level, up to the Hilton’s white line, the rectal epithelium is a non-keratinized, pluristratified pavement. In the area of interference between the two territories, histologists describe a state of “cellular unrest”, which predisposes to phenomena of metaplasia and even malignant degeneration. Gland structures with different morphology and function can contribute to oncogenesis [23].

### 3.4. Environmental Factors

The geographical distribution of colorectal cancer is uneven between different countries or continents. The migration of some population groups to regions with high incidence increases the frequency of this pathology, suggesting the influence of some environmental factors [24].

### 3.5. Age and Gender

The distribution of colorectal cancer between the two genders is relatively equal, with the prevalence on the right colon in women and on the left colon in men. The maximum incidence is recorded at 60–70 years of age, although it is more and more common at a young age [25]. In the case of obstructive colorectal cancer, the proportions are also similar, with various studies showing heterogeneous results, some of them highlighting a slightly higher frequency in men and others in women [26,27,28,29].

### 3.6. Precancerous Colorectal Disorders 

Inflammatory bowel diseases (Crohn’s disease, ulcerative colitis) and diffuse colic polyposis, register a significant percentage of malignant degeneration in the absence of treatment or in conditions of insufficient or incorrectly conducted treatment [24,30]. The risk of colorectal cancer may vary between 0.06% and 0.2% reported annual incidence, between 2.5% and 8% reported cumulative incidence of 20 years, and between 7.5% and 18% reported cumulative incidence of 30 years of inflammatory bowel disease [31,32,33]. 

### 3.7. Diet

The low intake of vegetables and cellulose fibers and the excess of animal fats, carbohydrates, and alcohol predisposes to the development of colorectal neoplasia [34].

### 3.8. Hepatobiliary Disorders and Cholecystectomy

The excessive presence of secondary bile acids (deoxycholic and lithocholic) in the digestive tract promotes carcinogenesis. The effect seems mediated by the excessive fixation of dietary calcium, which causes punctual peeling of the cholic mucosa, favoring the appearance of metaplastic changes. Large bile discharges into the digestive tract post-cholecystectomy raised the suspicion that this surgery predisposes to colorectal cancer [35,36].

### 3.9. Various Factors

Obesity, sedentary lifestyle, smoking, diabetes, abdominal radiotherapy, racial factor, etc., are other factors that can leave their mark on colorectal carcinogenesis [37,38,39,40,41].

### 3.10. Drug-Related Factors

Vitamins A, C, E, beta carotene, aspirin, and non-steroidal anti-inflammatory drugs seem to have a protective role, noting a higher incidence of colorectal neoplasm in those with vitamin deficiencies [42,43].

## 4. Development of the Obstructive Mechanism 

The spontaneous evolution of colorectal cancer is always burdened by complications. Their variety is very large. Among the local complications, the most common are: loco-regional invasion with possible inter-visceral or external fistulas, tumor infection with adjacent sclerolipomatous reaction or abscesses, peritonitis by evacuation of tumor abscesses or intestinal perforations, intestinal obstructions, lower digestive bleeding, etc. General complications are represented by anemia, paraneoplastic venous thrombosis, multiple metastases, hepatic abscesses with systemic sepsis, etc. [44,45].

The most common complication is low bowel obstruction, found in approximately 20% of the cases of colorectal cancer and can occur either relatively abruptly, or is preceded by initially discrete premonitory symptoms, non-specific (until advanced evolutionary stages), and generally neglected or incorrectly interpreted [18,46].

The common obstructive mechanism is simple obstruction and is specific to situations with preceding manifestations (transit disorders, anemia, weight loss, etc.). For forms with sudden onset may occur intussusceptions (rare at the colic level and non-existent at the rectal level), volvuluses of supratumoral mobile segments and the association of peritoneal carcinomatosis or enteral ischemic phenomena. Colic volvulus is favored by the increased weight of endoluminal content and hypermobility, with fixed points of the extremities as close as possible. In intestinal volvuluses, necrosis is not directly correlated with the number of rotations of the loop, but with the degree of “constriction of the affected mesenteries” [29,47].

Low bowel obstruction due to neoplastic cause (colon and rectal cancer) presents anatomical and functional characteristics important in stage, pathogenic, topographic, and etiological diagnosis, orienting therapeutic consequences.

### 4.1. Anatomical Factors

#### 4.1.1. The Diameter of the Colorectal Segments

The simple obstructive mechanism is installed later in colic segments with large caliber. Subsequently, the ileocecal valve follows the segment with the largest diameter (the cecum and the ascending colon), which explains the lower frequency of right colon occlusions if the Bauhin valve is not interested. The diameter of the colic lumen decreases discreetly to the distal segments (except the rectal ampulla). Peritumoral sclerolipomatosis and anemia are more common in the right colon, contributing to the shaping of the characteristics specific to this level of tumor location [48,49].

#### 4.1.2. The Thickness of the Colic Wall 

**Figure 1:**
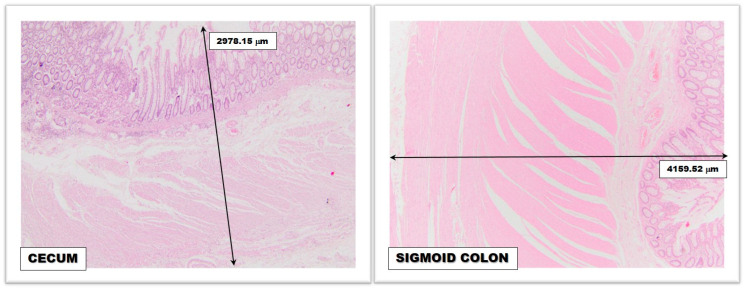
Microscopic image revealing the difference between the thickness of the normal cecal wall and the normal sigmoid colon wall; magnifying glass, 4× (obtained from the Histopathology Department, “Bagdasar-Arseni” Clinical Emergency Hospital from Bucharest).

The thickness of the colic wall has less importance regarding the frequency of colorectal neoplasms on certain topographies but has profound relevance in relation to retrograde diastatic perforations (Laplace’s law) (Figure 1). The large colic caliber associated with the reduced thickness of the wall allows the development of lateral rupture pressure that makes the cecum and ascending colon wall vulnerable (the rupture pressure at this level is approximately 80–100 cm H_2_O, compared to the one in the small intestine where it is approximately 200–300 cm H_2_O) [50,51,52,53].

#### 4.1.3. Colorectal Vascularization

Although it enjoys two important arterial sources (upper mesenteric artery for the right colon and lower mesenteric artery for the left colon) and marginal arches Drummond and Riolan-Haller and inconstant central intermesenteric anastomosis Huard, the colon does not benefit through long and short vasa recta arteries of a generous arterial flow comparable to the upper segments of the tract digestive system (stomach and small intestine). Mesenteric arteries are the preferred territories for the phenomenon of atheromatosis contributing to the progressive decrease of blood flow in irrigated territories. Elongation of vasa recta in the distended colic wall favors ischemia and parietal microthromboses, precipitating colic perforation. The closing of the venous network (a consequence of parietal pressure) is an additional factor in the irreversibility of parietal lesions [54,55].

#### 4.1.4. Anatomical and Functional Sphincters 

The colorectal area stretches between the ileocecal valve and the anal sphincter. The latter is of limited importance in low intestinal obstruction, being most commonly located under the obstacle. The ileocecal valve is deeply involved in the pressure play and dynamics of the cholic stasis content in low intestinal obstruction. A “permissive” valve can favor the reflux of stercoral content towards the ileal and then jejunal territory, tempering for the moment the axial and lateral pressures from the obstructed colon. Its increased pressure competence can close the possibility of retrograde reflux, with the exponential increase of intracolic pressures.

The functional sphincters of the colon (Cannon-Boehm, Payr, Moutier, and Obiern) are limited areas with better represented circular musculature and may have a limited role in colon dynamics in the early stages of the obstruction evolution. It can be concluded that Laplace’s physical law may be influenced by cholic morphofunctional characteristics [56,57].

#### 4.1.5. The Mobility of Colic Segments 

The mobility of colic segments can influence the dynamics of the obstructive process. If the fixed segments are the site of tumor development, they will suffer a simple process of obstruction, while for mobile segments other obstructive mechanisms may overlap (intussusceptions, volvuluses, etc.) [58].

#### 4.1.6. Tumor Morphopathology

**Figure 2:**
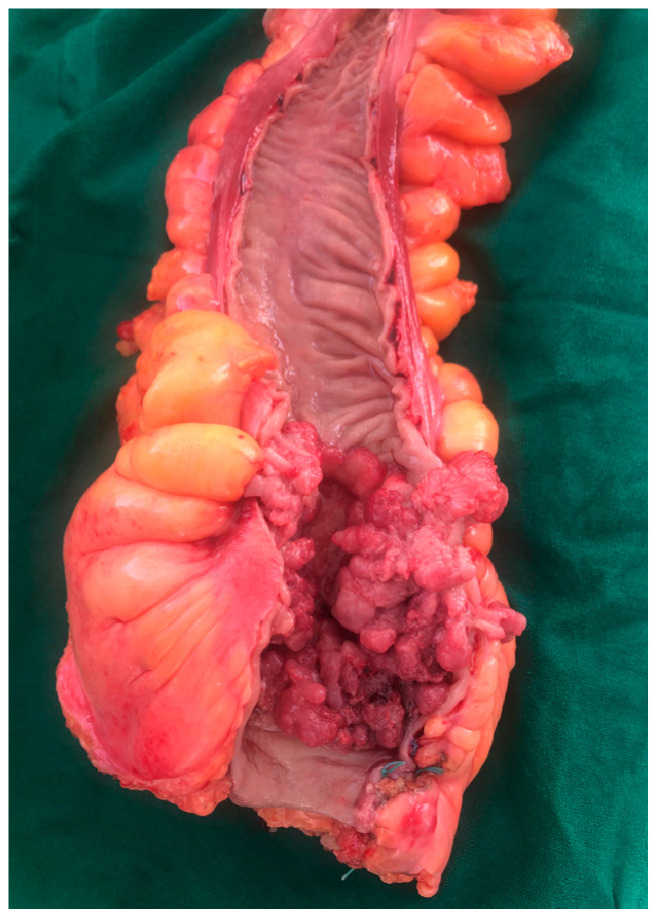
Postoperative specimen of a tumor located in the colorectal junction (collection of General Surgery Department—“Bagdasar-Arseni” Clinical Emergency Hospital from Bucharest).

Obstruction of the colic lumen is more common in ulcer-vegetative (Figure 2), exophytic, and polypoid forms. Diffuse infiltrative forms can also be incriminated, especially in situations where the axial extension of the tumor is very large [59]. 

### 4.2. Functional Factors

#### 4.2.1. Colic Peristalsis

Colic innervation is vegetative, receptor and effector. The Meissner submucous plexus and axons of neurons in the posterior root node of the spinal nerves T11-L1 are responsible for visceral sensitivity. The secretive and motor activities are modulated by the Auerbach myenteric plexus, unequally represented for the right colon (related to the upper mesenteric plexus) and the left colon (dependent on the lower mesenteric plexus). The poor representation of the myenteric plexus is associated with a diminished peristaltic accompanied by a background distension of the left colon (megadolichocolon), decompensated early in the obstacles of colorectal junction or below this level. Colic hyperdistension makes the action of pharmacological active factors (acetylcholine, neostigmine) ineffective on tonus and peristalsis [60,61].

#### 4.2.2. Other Functional Factors 

Other functional factors involved in the pathogenic mechanisms of low intestinal obstruction depend on the individual characteristics of each patient, or derive from anatomical considerations: cholic resorptive function, dramatically extracted dysmicrobisms [62,63], nutrition prior to the installation of the obstruction, degree of damage to the mucus-epithelium barrier [64], associated diseases that amplify the effects of dysvolemia and sepsis, various reflex factors, etc. [65,66].

Success in the complex treatment of a low neoplastic obstruction is conditioned by a complete diagnosis, adequate pre-operative preparation, a surgical act adapted to the case (in one, two or three successive stages) and dynamic postoperative care. Diagnostic errors consist both in overestimating the case (sometimes practicing exploratory laparotomy in medical conditions that evolve with intestinal paresis), but especially in the negative (assessment of the case as a non-surgical emergency, failure to identify cases with initial ischemic mechanism, not performing intraoperatively the diagnosis of all obstructive mechanisms involved and incomplete, inadequate or with potential for relapse surgical solutions). Etiological clarification is only possible sometimes, but this aspect is not a major drawback given the indication of surgical exploration, which will clarify this aspect as well.

Preoperative management of patients diagnosed with bowel obstruction involves, even from the emergency room, the placement of a peripheric venous catheter and starting the infusion of crystalloid solutions such as saline or Ringer solution. Acid-base and electrolyte rebalancing is a priority, often requiring repeated assessments of serum ions and pH. Considering the potential for dehydration and the rapid evolution of the disease, a urinary catheter is necessary for monitoring diuresis. In patients with severe cardiac, renal, or pulmonary failure, monitoring of fluid and electrolyte rebalancing using central Swan-Ganz catheters may be helpful. Preoperative hematological imbalances such as severe anemic syndromes can be adjusted by blood transfusions and severe thrombocytopenia by administering platelet masses. In the case of coagulopathy (liver cirrhosis, hematological disorders) or in case of changes in coagulation parameters due to treatments for various cardiologic disorders, prompt administration of plasma or vitamin K is necessary. Antibiotherapy, thromboembolic prophylaxis, and treatment of associated diseases are equally important [52,67,68,69,70].

The moment of surgery should be chosen with great care and is the result of the experience of the anesthetic-surgical team. The operative act must be adapted to the case and has as its main objective the resolution of intestinal obstruction and only in a secondary way the resolution of the generating disease. The resection of the involved colorectal segment is performed (right or left colectomy, segmental colectomy, rectocolectomy), respecting oncological principles. Afterward, anastomosis is taken into account when the proximal segment shows no structural changes. Otherwise, to avoid a digestive fistula, it is recommended to close the distal segment and perform an ostomy at the level of the proximal one [71].

Often, the surgical procedure does not involve the removal of the obstructive process, but only the restoration of the digestive transit. This involves cases in which is intended to shorten the duration of the surgical intervention due to the patient’s comorbidities and the general condition at the time or procedures performed for palliative purposes, for advanced neoplasia. In the case of external digestive derivations without removal of the obstructive process, it is recommended to perform a continuous ostomy, in order to avoid a “closed loop” [72].

“The patient with intestinal obstruction is in the situation of a rescued from drowning. This is not the case for a swimming lesson”—Wangensteen.

The postoperative stage can crown the effort made to save the patient or compromise the previous efforts and should be managed by a multidisciplinary team. Analgesic, antibiotic, prokinetic, and anticoagulation therapy in the case of prolonged immobilization should be taken into account. Diuresis and intestinal transit will be monitored, and oral nutrition will be gradually resumed. Local complications (wound abscess, necrosis, evisceration, ostomy dehiscence or parastomal abscess) or general complications, such as cardiorespiratory, renal, or hepatic failures, should be closely monitored and treated promptly [73].

## 5. Conclusions

○ The main cause of low bowel obstruction is colorectal cancer.
○ Preceded by early or sudden signs, low neoplastic obstruction generally has the meaning of a neoplasm in an advanced evolutionary stage.
○ The therapeutic measures adopted (medical-surgical) must have a dynamic character in accordance with the particular situation of the patient.
○ Except for certain or probably benign etiologies, the possibility of colorectal neoplasia should always be considered, in low obstructions, regardless of the patient’s age.
○ A “truce” in the fight against the obstruction can be deceptive.

## Data Availability

Data is unavailable due to privacy or ethical restrictions.
